# Thermal signatures of voluntary deception in ecological conditions

**DOI:** 10.1038/srep35174

**Published:** 2016-10-13

**Authors:** Maria Serena Panasiti, Daniela Cardone, Enea F. Pavone, Alessandra Mancini, Arcangelo Merla, Salvatore M. Aglioti

**Affiliations:** 1 Department of Psychology, University of Rome “La Sapienza”, I-00185 Rome, Italy; 2 IRCCS, Fondazione Santa Lucia, I-00179 Rome, Italy; 3 Institute for Advanced Biomedical Technologies, G. d’Annunzio University, Chieti and Department of Neuroscience, Imaging and Clinical Science, University of Chieti-Pescara, I- 66013 Chieti, Italy

## Abstract

Deception is a pervasive phenomenon that greatly influences dyadic, groupal and societal interactions. Behavioural, physiological and neural signatures of this phenomenon have imporant implications for theoretical and applied research, but, because it is difficult for a laboratory to replicate the natural context in which deception occurs, contemporary research is still struggling to find such signatures. In this study, we tracked the facial temperature of participants who decided whether or not to deceive another person, in situations where their reputation was at risk or not. We used a high-sensitivity infrared device to track temperature changes to check for unique patterns of autonomic reactivity. Using a region-of-interest based approach we found that prior to any response there was a minimal increase in periorbital temperature (which indexes sympathetic activation, together with reduced cheek temperature) for the self-gain lies in the reputation-risk condition. Crucially, we found a rise in nose temperature (which indexes parasympathetic activation) for self-gain lies in the reputation-risk condition, not only during response preparation but also after the choice was made. This finding suggests that the entire deception process may be tracked by the nose region. Furthermore, this nasal temperature modulation was negatively correlated with machiavellian traits, indicating that sympathetic/parasympathetic regulation is less important for manipulative individuals who may care less about the consequences of lie-related moral violations. Our results highlight a unique pattern of autonomic reactivity for spontaneous deception in ecological contexts.

Classic studies based mainly on standard polygraphic recordings indicate that lie-telling is accompanied by changes in the autonomic system’s activity^1^,^2^,^3^. Yet it remains unclear whether any specific pattern of autonomic reactivity can be linked to deceptive behaviour. Moreover, standard techniques for recording autonomic activities imply positioning of intrusive electrodes that might lower the ecological validity of deception experiments. Thermal infrared (IR) imaging allows researchers to circumvent this problem by estimating cutaneous temperature (and its subtle changes) via a contact-free technology that records autonomic nervous system responses. The importance of thermal imaging has been demonstrated by studies which show that different sympathetic activities can be deciphered from the distinct facial temperature patterns elicited by different psychological or physical states^4^,^5^,^6^,^7^,^8^,^9^. Warming in the periorbital regions, for example, seems to reflect activation of the sympathetic nervous system related to flight-or-fight. Nasal skin temperature can reflect increased sympathetic activity with vasoconstriction and drop in skin temperature: this has been seen both in monkeys^10^,^11^ and humans^12^,^13^,^14^. Similarly, reduced sympathetic activity may cause nasal vasodilatation and heightened skin temperature^15^,^16^. It is also worth noting that rising cheek temperature may reflect sympathetic activity related to blushing reactions^17^ caused by embarrassment feelings^18^, while decreasing cheek temperature occurs in response to startle stimuli^19^^ ^. Nasal and perioral temperature variations have been shown as responses to social stimuli of moral valence in children and empathic adults^8^,^9^,^20^. IR has already been applied to lie detection^19^,^21^,^22^,^23^,^24^,^25^. In a seminal study, Pavlidis and collaborators^19^ asked participants to commit a mock crime and then testify to their innocence. They found that warming around the eyes was a good index of deception. Tsiamyrtzis and collaborators^24^ then found that warming around the eyes signalled deception 87.2% of the time. According to Pollina and collaborators^23^, the lie-related warming effect was more pronounced in the right hemiface. A 98.89% classification rate of guilty and innocent participants was reached by Park and collaborators^21^ by using a functional discriminant analysis of temperature in the periorbital areas. Finally, Zhu and collaborators^26^ found that the thermal forehead signal enabled a 76.3% success rate in deceptive state classification. It is worth noting that changes in periorbital and forehead area temperature are directly related to stress level^27^,^28^: increased blood flow around the eyes may facilitate rapid eye movements during preparedness for flight^4^, while increased forehead temperature may indicate activation of the corrugator muscle, which is highly correlated with mental stress^29^.

However, experimental paradigms of past thermal studies were not always completely ecological: asking participants to pass a lie detection test after having committed a mock crime cannot elicit the same moral conflict of everyday life deception. Indeed, both the crime and the lies were required by the experimenter in most of the above circumstances, thus precluding any personal decision making process. The warming in periorbital regions detected by these paradigms is believed to be a sign of anxiety^4^ triggered during the lie detection task by the fear of being caught. Importantly, besides this fear, moral decisions are often emotionally charged^30^. According to the Somatic Marker’s Hypothesis, emotion (a collection of changes in body and brain states triggered by the specific contents of one’s experienced or recalled perception) guides decision making by imbuing behavioural options with affective valence. Relevant to the current study is that emotional deficits are frequently present in pathologies such as antisocial personality disorders or the ventromedial prefrontal cortex lesions associated with anomalous moral behaviour^31^,^32^. So while the role of emotions in moral decisions has been extensively studied (for a review see ref. 33), only one study, as far as we know, has attempted to explore the role of emotions in real (and not instructed or sanctioned) moral decisions^34^. Social psychology has shown that deception varies across different individuals^35^,^36^,^37^,^38^ and situational circumstances^35^,^39^,^40^,^41^,^42^, but no study has attempted to explore the influence of these factors on the autonomic correlates of deception.

In the present study we used facial thermal imaging to investigate the role of the autonomic system in deceptive decision making. To determine whether any thermal signature of lying is modulated by dispositional and situational variables, we used the Temptation to Lie Card Game (TLCG)^35^,^43^, an ecologically validated paradigm in which participants are free to lie to their opponent about the outcome of a choose-a-card game (a schematic representation and description of the experimental task is shown in Fig. 1). Throughout the experiment, thermal infrared imaging allowed us to measure facial skin temperature in periorbital areas as well as cheek and nasal tip areas. Using this region of interest (ROI) approach we obtained a complete pattern of facial reactivity and a fine-grained picture of the thermal autonomic correlates of deception.

We hypothesized that i) the moral conflict related to spontaneous deception would elicit a thermal facial pattern different from the one highlighted by previous studies in which deception was allowed and sanctioned by the experimenter; ii) the decision to deceive per se would be accompanied by an emotional activation, even if participants did not risk to be punished for their behavior; iii) and finally, based on our previous findings^35^,^43^, that dispositional traits (such as manipulativeness, moral disengagement or impression management) and situational circumstances (such as reputational risk) would modulate the thermal autonomic correlates of deception differently.

## Results

### Behavioural data

The number of lies per condition were entered in a 2 × 2 ANOVA with Outcome (Self-gain vs. Other-gain) and Reputation (Reputation Risk vs. No-Reputation Risk) as within-subject factors. We found a significant main effect of the Outcome F(1, 11) = 22.46, p = 0.0006, bootstrap p = 0.0002 and a significant interaction Outcome x Reputation Risk F(1, 11) = 4.23, p = 0.06, bootstrap p = 0.038) (Table 1).

We found a decrement in self-gain lies (p = 0.02) in the risk for reputation condition but not in the no-risk for reputation condition. No such effect was found for other-gain lies (p = 0.34). These results are very similar to those of our previous studies^35^,^43^.

### Thermal imaging data

We entered normalized to fixation temperature values (see the methods section) into three separate ANOVAs (one for each ROI) with Type of Response (Self-gain Truth, Self-gain Lie, Other Gain Truth), Reputation (Reputation Risk, No Reputation Risk), and Trial Phases (Card Positioning, OP’s choice, Response Preparation, Post Response, Feedback) as main effects. Other gain lies were excluded from the analysis because participants did not produce enough responses of this type.

#### Cheeks

**Figure 2:**
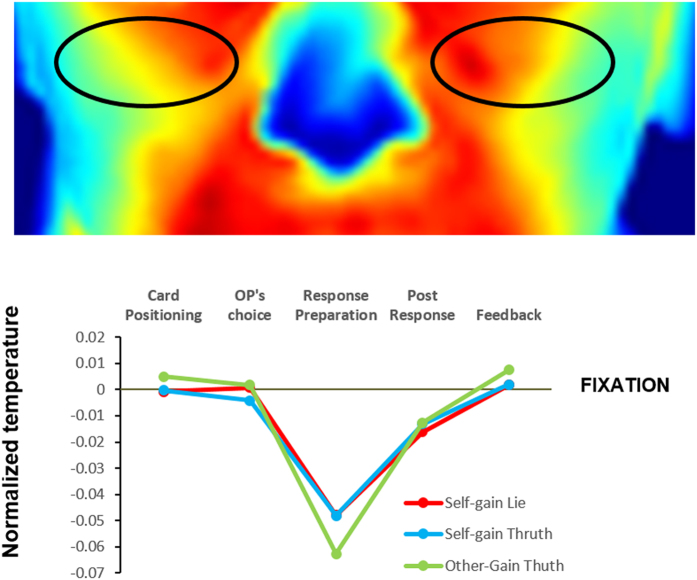
Temperature of the cheeks (normalized with respect to the fixation) for the five experimental phases. A significant decrease during response preparation (Phase 3) is found. The double interaction Type of Response x Phases did not show any significant post hoc comparison.

The ANOVA we conducted on cheek areas showed a significant main effect of Trial Phases F(4, 44) = 5.28, p = 0.001 ηp^2^ = 0.32, bootstrap p = 0.002 . The colours of the template display the temperature of one representative participant during the fixation phase. Post-hoc comparisons indicated that the temperature of these areas was cooler during the Response Preparation (Phase 3) than in all other conditions (all ps < 0.01, Bonferroni corrected). We also found a significant interaction of Type of Response x Trial Phases F(8, 88) = 2.33 p = 0.02, ηp^2^ = 0.17, bootstrap p = 0.002. Importantly, however, post hoc comparisons showed that the interaction comparisons within each phase did not contribute to the significance. More specifically, the Response Preparation phase’s low temperature was found in all type of responses (Fig. 2).

#### Periorbital regions

**Figure 3:**
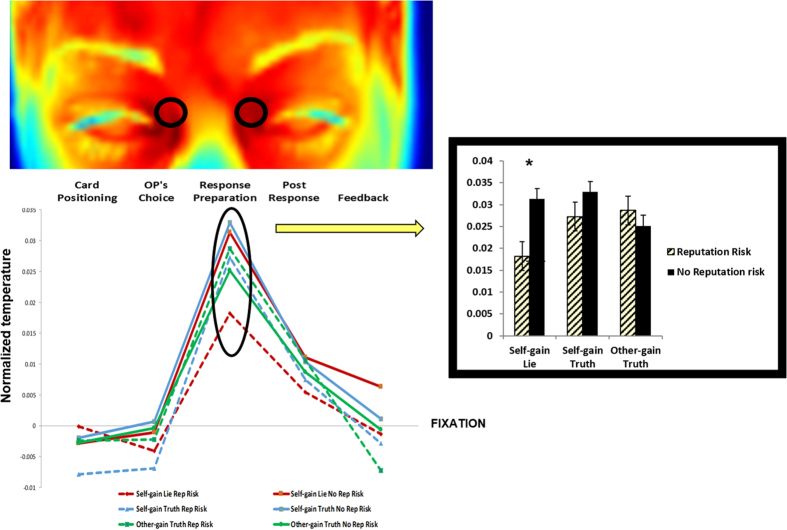
Temperature of periorbital areas (normalized with respect to the fixation) for the five experimental phases. The colours of the template display the temperature of one representative participant during the fixation phase. Periorbital areas showed a significant warming during response preparation (Phase 3) (all ps < 0.01). This warming was weaker for the Self-gain lies in the reputation condition (significant triple interaction, bootstrap p = 0.04; Bonferroni corrected post hoc Self-gain lies Reputation Risk vs. No Reputation Risk, p < 0.05).

The ANOVA of temperature values in the periorbital areas showed a significant main effect of Trial Phases F(4, 44) = 13.66, p < 0.0001 ηp^2^ = 0.55, bootstrap p = 0.0002 which was entirely accounted for by a warming in the Response Preparation (Phase 3) (all ps < 0.005, Bonferroni corrected). The interaction of Type of Response x Trial Phases x Reputation was significant F(8, 88) = 1.92, p = 0.065, ηp^2^ = 0.14, bootstrap p = 0.04) and was explained by diminished warming during Response Preparation to Self-gain lies in the Reputation Risk condition compared to the No Reputation Risk condition (p < 0.05) (Fig. 3).

#### Tip of the nose

**Figure 4:**
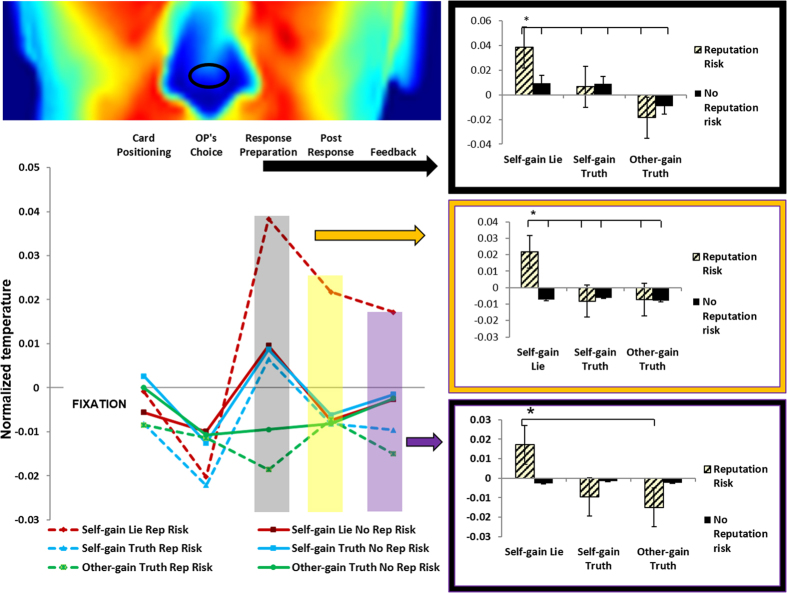
Temperature of the tip of the nose (normalized with respect to the fixation) for the five experimental phases. The colours of the template display the temperature of one representative participant during the fixation phase. The tip of the nose showed a selective increment in temperature for Self-gain lies in the reputation condition but not in the other responses during response preparation (all ps < 0.03) and the post response phase (Phase 4) (all ps < 0.05). The feedback phase from the same condition (Phase 5) did not differ from the response preparation phase and the post response phase (all ps > 0.05), but it was different from the feedback phase of the other-gain truth (p < 0.01).

This ANOVA showed a significant three-way interaction Type of Response x Trial Phases x Reputation F(8, 88) = 2.03, p = 0.05, ηp^2^ = 0.16; bootstrap p = 0.03. Post-hoc tests showed that tip of the nose temperature, when participants produced Self-Gain Lies in the Reputation Risk condition, was higher in the Response Preparation than in all the other phases and conditions (all ps < 0.03, Bonferroni corrected), except for the Post Response and the Feedback phases of the same condition. These results indicate that tip of the nose warming began during the preparation phase and lasted until the feedback was given. In addition, nose temperature in Post Response for Self-Gain Lies was higher in the Risk Reputation condition than in the other Post-Response (all ps < 0.05, Bonferroni corrected) conditions. In contrast, tip of the nose warming was significantly higher in Feedback for Self-Gain Lies in the Reputation Risk condition when compared to Other-Gain Truths in the reputation risk condition (p < 0.01, Bonferroni corrected), but not in any other condition (all ps > 0.05, Bonferroni corrected) (Fig. 4).

### Correlations between thermal imaging data and personality traits

**Figure 5:**
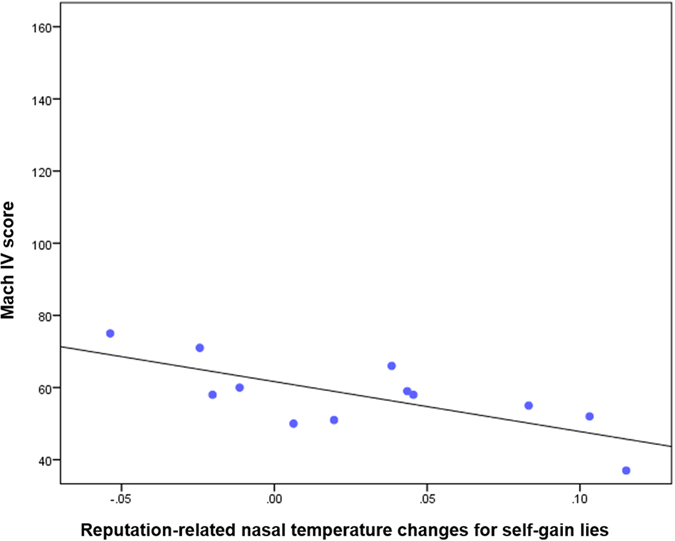
Correlation between Reputation-related nasal temperature changes for self-gain lies and Machiavellianism traits. The above index was calculated by subtracting the nasal temperature when producing self-gain lies in the no reputation risk from reputation risk condition in the response preparation phase. The scatterplot shows a significant negative correlation (r = −0.724, p = 0.008).

We observed a significant increase in tip of the nose temperature for Self-Gain Lie choices in Response Preparation, Post Response, and Feedback phases. We correlated (separately for each of these phases) personality traits with the nose temperature for Self-Gain Lies in Reputation vs No-Reputation Risk. While no correlation was found in the last two phases, a significant negative correlation was found between this index for Response Preparation and MACH IV (r = −0.724, p = 0.008). These findings indicate that the more participants were manipulative, the less sensitive they were to the reputation risk condition when performing self-gain lies. The requested level of significance was adjusted by dividing it by the number of comparisons for each phase (0.05/4 = 0.0125) (Fig. 5).

## Discussion

By using high resolution thermal infrared imaging, we investigated the autonomic reactivity reflected in changes of facial temperature contingent upon the voluntary decision to produce lies. Different from previous studies, we did so in ecological—if highly controlled— laboratory conditions. Moreover, we explored whether changes in the decision to lie for personal advantage were modulated by dispositional (e.g. personality traits) and situational (e.g. reputational risk) variables. We used a modified version of our TLCG, a validated card-game paradigm in which participants are tempted to lie to another person while remaining free to do so or not^35^,^43^. In the reputation risk condition, participants were under the impression that their truth or lie would be known by the opponent player; in the no reputation risk condition they were told that the opponent would be ignorant of this information. In keeping with our previous studies^35^,^43^, the behavioural results indicate that self-gain lies were less frequent during the reputational risk condition. The results also confirm that the experimental paradigm is adept at catching situational determinants of the decision to lie. To explore autonomic reactivity we made an analysis of lie-related changes in facial temperature that was based on the definition of regions of interest. This approach revealed a general pattern of facial reactivity related to the decision process and, more importantly, a unique pattern related to preparing and producing a lie in the risk-reputation condition.

As regions of interest we chose those facial regions that in previous studies have been involved in deception (periorbital areas), startling (cheeks and periorbital areas) and moral emotions like guilt (tip of the nose) (review in ref. 44). Note that periorbital vessels feed the main muscles around the eyes, which are known to be involved in startle reflexes. Importantly, the temperature increase in the periorbital areas that follows startling stimuli is paralleled by a temperature drop in the cheek region^6^,^28^,^44^. For example, concomitant periorbital warming and cooling over the cheeks were reported in stressful situations (e.g. in response startling stimuli^4^) and interpreted as a measure of one’s preparedness for flight.

Notably, the blood flow in facial vessels is regulated by the arteriovenous anastomoses which are highly concentrated in the nasal region. Nasal region temperature is thus considered a good index for both the activation and de-activation of the sympathetic system (which corresponds to a drop and a rise in temperature, respectively)^45^,^46^.

### Thermal indices of reactivity during preparation to choose between self vs. other and true vs. lie related options

Significant changes in periorbital and cheek region temperature were only found in the response preparation phase. Tellingly, changes in cheek region temperature were non-specific and were not influenced by the decision to lie or tell the truth (Fig. 3). This suggests that engagement of the sympathetic system that underlies the autonomic changes of reactivity in the cheek region may reflect the preparation to respond independently from the kind of decision ultimately taken (i.e. lying or telling the truth). In contrast, changes in periorbital region temperature were somewhat specific for the choice to lie. Indeed, there was only an increase in periorbital region temperature in the response preparation phase. Crucially, however, this increase was minimal for self-gain lie choice in the reputation risk blocks (Fig. 4). This result may reflect arousal deriving from the dilemma of whether or not to perform a moral violation when one’s own reputation is at risk. Note that lie-related autonomic reactivity in the periorbital regions was not found in the subsequent phases and thus does not seem to track the decision to implement a deceptive behaviour. Our results may seem at odd with studies showing that increase of periorbital temperature is a marker of deception^19^,^23^,^24^. However, this apparent contradiction may be explained by the difference between our paradigm and previous ones: in each of the studies cited above, participants were asked to commit a mock crime and then testify to their innocence during a lie detection task. Thus it is entirely possible that the crime related questions (in which participants had to successfully lie) triggered a fight or flight reaction related to the fear of being caught. In contrast, participants in our reputation risk condition always knew that their lie would be uncovered. This information might have induced the need to regulate the activation of their autonomic system in order to face the morally demanding situation of overtly acting in a dishonest manner.

### Thermal signatures of the decision to lie for self-gain when one’s own reputation is at risk

Importantly, we discovered one region, i.e. the tip of the nose, that seems to specifically track the decision to implement a self-gain behaviour at the cost of violating the moral code that prevents people stealing from others. On top of this, temperature changes in the nose region seem specifically related to one’s own reputation being at risk.

The increment in temperature on the tip of the nose is known to be related to a depression of the sympathetic system^15^,^16^. Interestingly, decreased sympathetic activity during deception was found by using transcranial direct current stimulation (tDCS) to inhibit the anterior prefrontal cortex (aPFC). Moreover, the inhibition was associated with a decreased feeling of guilt in situations of deception^47^. These findings suggest that the decision of whether or not to deceive another person generally activates the sympathetic system. Importantly, when reputation is at risk, participants need to downregulate the sympathetic system in order to engage in dishonest behaviour. A decrement in sympathetic activity is believed to reflect emotional control: it has been detected after the use of verbalization strategies^48^, the use of reappraisal technique during reward anticipation^49^, the viewing of unpleasant pictures^50^ and during decision making processes^51^. In particular, Sokol-Hessner and collaborators^51^ showed that the use of reinterpretation during a decision making task reduces loss aversion behaviour as well as the sympathetic response to loss. Thus we speculate that our participants felt the need to use some kind of emotion regulation strategy in order to be able to deceive in the reputational risk condition. Furthermore, we observed that the inhibition of sympathetic activity started before the response was made and lasted until the feedback was given. We believe that, in this condition, the feedback (namely: “You lied, you win”) acted as a reminder of the moral transgression. It has been shown that after engaging in deception, people tend to show “strategic forgetting” of moral rules^52^ as this allows them to reduce the cognitive dissonance between personal attitude (being honest) and actual behaviour (deception). This cognitive dissonance triggers a sympathetic reaction that is experienced as unpleasantly arousing^53^,^54^; thus it is entirely possible that our participants felt the need to subdue the sympathetic system in order to avoid the cognitive dissonance evoked by the provided feedback. One may ask why we only found this activation in the reputational risk condition. Our previous studies^35^,^43^ have already indicated that deception for participants is harder in the reputation condition, likely due to the heightened pressure to act in a socially appropriate manner. Similarly, Coricelli and collaborators^34^ found that tax evasion was discouraged less by monetary sanctions than by an environment where deception was made public. In line with our results, these authors also found that evaders, when informed that their identity would be revealed to the other players, displayed an initial increase in sympathetic activity (first trial effect) that was soon followed by a decrement. This effect suggests that participants learned to regulate their arousal in order to deceive during the public condition. It is also important to note that the difference between deception-related warming on the nasal tip in reputation risk and no reputation risk conditions was negatively correlated with manipulativeness traits. This means that the participants with higher Machiavellian intelligence exhibited less reduction in sympathetic activity while lying in the reputation risk condition. This finding accords with our previous results showing that manipulative people did not decrease their deceptive behaviour in the reputation risk condition^35^, nor did they show any inhibition in the cortical motor readiness to lie^43^. These people admit deceiving to get what they want^55^ and they feel little or no guilt while lying^38^. Thus, it is no surprise that participants with machiavellian intelligence in our study did not feel the need to suppress their sympathetic system while deceiving in the reputation condition. Overall, our results build on previous findings about the role of the autonomic system during deception. We showed that, generally, people who are *required to decide* whether to lie or tell the truth (and are not *required to deceive* in order to pass a lie detection test) have an active sympathetic system before responding, while an inhibition is required in order to lie when reputation is at risk. Although here we tested mainly females, the current behavioral results are identical to those of our previous studies^35^,^43^ where males and females were balanced. However, even if gender does not influence the lie behaviour in our task, its influence on the autonomic correlates of spontaneous deception should be addressed by future studies. At any rate, our results shed light on the critical role that personality differences may play in the autonomic correlates of deception, and suggest that these differences should be taken into account when investigating such complex behaviour.

## Methods

### Participants

Nineteen participants were recruited for the study. Two of them were excluded from further data analysis because they never lied during the experiment; two did not believe they were playing against another player; three were excluded for technical problems. Thus, we analyzed data from twelve participants (8 females, age range 18–32, mean 26.7). The inclusion criterion was absence of any overt physical, psychiatric or psychological disease. All participants were asked to i) refrain from heavy physical activity and the intake of vasoactive substances (i.e. caffeine, nicotine) for two hours prior to the measurements; ii) be clear of cosmetic substances on their faces at the time of the experiment^44^,^56^. The experimental protocol was approved by the ethics committee of the Fondazione Santa Lucia and was carried out in accordance with the ethical standards of the 1964 Declaration of Helsinki. Written informed consent was obtained from all participants after full explanation of the study procedure.

### The temptation to Lie Card Game

In the TLCG, an opponent (OP) has to pick one of two covered cards and the participant has to communicate the outcome of the choice to the OP (i.e., win or lose). Unbeknown to the participant, the OP’s choice is made by a computer. Participants were told that previous research had shown that the physical features of others could influence social decision-making, and that because of this they would only meet the OP at the end of the experiment. Participants were also told that the OP was seated in a different room and that no information about the OP (e.g., gender, age) could be provided until the end of the interaction. This procedure was adopted because we previously demonstrated that the physical presence of the OP does not affect participants’ performance on this task^35^. Only at the end of the experiment were participants fully debriefed and told that the OP’s choices were made by a computer algorithm.

Participants were instructed that the OP’s task was to choose one of two covered cards, the ace of hearts or the ace of spades. The former and the latter indicated gain and loss for the OP, respectively. The OP could not see the outcome of the choice, which was communicated verbally to him/her by the participant. By lying, the participant had the chance to reverse the outcome of the game: winning when he/she had actually lost (self-gain lie) or losing when he/she had actually won (other-gain lie). Trials in which the outcome of the OP’s choice implied a gain or loss for the participant were defined as favorable and unfavorable outcomes, respectively. Participants performed the game in 2 blocks: reputation risk (R), in which they knew that the OP was informed about their choice to lie or tell the truth; and no-reputation risk (NR), in which they knew that the OP was ignorant of the choice. The order of the 2 conditions was counterbalanced across participants. Both players had 25 euros with which to play. In each trial, the winner took money from the other player. Participants were told that a different amount of money was associated with each trial and that the exact gain would only be communicated at the end of the game. This procedure allowed us to: i) rule out the possibility that participants’ behavior was based on a trial-by-trial computation of gain and loss and ii) ensure that the temptation to deceive was comparable between trials (the participants knew that each trial could be associated with a high monetary reward, and thus the temptation to deceive was largely fixed). Participants were paid 10 euros for their participation and had the possibility to win up to extra 25 euros, during the game.

### Procedure

Prior to testing, each subject was left in the experimental room for 20 minutes in order to allow the baseline skin temperature to stabilize^44^. The recording room was set at standardized temperature (23 °*C*) and humidity (50–60%) by a thermostat. Participants sat comfortably on a chair during both acclimatization and measurement periods. Stimulus presentation, timing and randomization were controlled using E-Prime ver.1.2 software (Psychology Software Tools Inc., Pittsburgh, PA) that was run on a PC. Participants sat 100 cm away from the thermal camera. The experimental stimuli (two play cards consisting in an ace of heart and an ace of spades) were projected onto a projector sheet located approximately 300 cm from the subjects. Each trial started with the presentation of a central fixation cross that lasted 1000 msec, followed by the presentation of the stimuli. The left/right position of the heart/spades ace was counterbalanced. After a varying time interval (between 2000 and 3000 msec), one of the two cards became bigger, indicating the OP’s choice. This randomized interval was employed so that the OP would seem like a real person. After each OP’s choice, participants were asked to report the game’s outcome to the OP through a microphone, saying either “Hai Vinto” (Italian for “You won”) or “Hai perso” (Italian for “You lost”). The stimulus remained visible on the screen until the response was given. After each trial, subjects received a feedback lasting 5000 msec that indicated their answer as well as the game’s outcome (e.g. “You lied, you won”; “You told the truth, you lost”). Participants were told that the feedback was delivered by a computer algorithm; it was actually delivered by an experimenter who could hear the participants’ responses. Each block contained 40 trials; half provided the Unfavourable outcome (the OP won) while the other half provided the Favourable outcome (the OP lost). Pauses were given every 20 trials.

### Manipulation check

Participants completed a nine-item questionnaire immediately after the experiment. In order to avoid directly asking participants whether they believed in the interaction (for such a question could trigger doubts about the veracity of the experimental situation), we created a cover story in which we described the questionnaire as designed to investigate their impressions on the other player, and whether or not these impressions might have influenced the interaction. The following questions were asked: 1) How lucky do you think your opponent was during the first match? 2) How lucky do you think your opponent was during the second match? 3) Do you think your opponent used a pre-defined strategy? 4) During the game, did you picture the opponent as a person as old as you? 5) During the game, did you picture the opponent as someone of your gender? 6) Do you think your opponent is angry with you now? 7) Did you use a pre-defined strategy?

The last two questions were target questions used to measure the strength of participants’ belief in the interaction:

8) Despite not playing in the same room as your opponent, did you feel involved in the interaction? 9) How involved did you feel in the game?

Thus, the first seven questions acted as cover questions, while 8 and 9 were target ones. Evaluations along a 5-point Likert scale (ranging from 1 to 5) were required for questions 1, 2 and 9. For questions 3, 4, 5, 6 and 7, a “yes”, “no” or “I don’t know” response was required. Question 8 required a mere “yes” or “no” response. The two subjects who responded “no” to item 8 were excluded from the analysis.

#### Personality measures

After the manipulation check, participants were administered the Balanced Inventory of Desirable Responding (BIDR) scale, which is composed by two 20-item subscales (each ranging from 20 to 140 score)^57^. BIDR measures two components of social desirability (i.e. self-deception and impression management); the Machiavellianism Scale (MACH IV), a 20-item scale where scores can range from 40 to 160, measures the ability to use deception and manipulation to acquire power during everyday life interactions^55^; and the Moral and Civic Disengagement (MD2) scale, a 40-item questionnaire (scoring from 40 to 200) measures an individual’s tendency to make use of self-exonerative manoeuvres when violating civic obligations in order to soften the moral consequences of their behaviours.

### Thermal imaging data acquisition and analysis

Thermal imaging was performed by means of a digital thermal camera (FLIR SC3000, FlirSystems, Sweden), with a Focal Plane Array of 320 × 240 QWIP detectors capable of collecting the thermal radiation in the 8–9 *μm* band with a 0.02 second time resolution and 0.02 *K* temperature sensitivity. Sampling rate for thermal imaging was set at 10 frame/sec. As regions of interest (ROI) we selected periorbital areas, nasal tip and cheeks (areas were defined according to^7^,^22^,^58^,^59^,^60^,^61^.

Data were down-sampled according to the experimental phases, meaning that for each trial we selected six images: the last image recorded when participants were observing the fixation cross (Phase 0-fixation); the last image recorded when participants were observing the two cards position (Phase 1-Cards positioning); the first image recorded when participants were observing the opponents’ choice (Phase 2-Choice of the opponent); the image recorded immediately before subjects’ response (Phase 3-Response Preparation); the image recorded immediately after subject’s response (Phase 4-Post-Response); the image recorded at the end of the feedback presentation (Phase 5-Feedback).

These images were chosen in order to capture either phasic or preparatory and tonic prolonged thermal responses. To objectively assess variations in facial temperature distribution, we registered each frame onto a common template according to the warping procedure proposed by Goshtasby^60^,^61^. We adopted the same processing method described in ref. 62; (for a detailed explanation see the APPENDIX A in ref. 62). The aim of image registration is to perform a spatial normalization of images representing regions of interest, i.e. the face, at intra-individual (i.e., among different frames) and inter-individual (among participants) levels. Spatial normalization involves warping-based transformation of the thermal images from a variety of individuals or frames into a common anatomical space (template). This was done so that changes in the temperature distributions could be compared on a pixel-to-pixel basis. After having down-sampled the time series of the facial thermal images for each participant, we registered each segmented image to the reference anatomical template to create a time series of registered (warped) images. We used average temperature time series for each ROI for the statistical analysis. The warping procedure ensured a reliable ROIs’ positioning and sizing, as every single thermal image was registered on a common template. We adopted the following criteria in order to ensure a reliable positioning and sizing of the ROI: for the nasal tip, the ROI was a circular region placed over its center that did not extend beyond the nostrils; for the periorbital ROI we used a circle with the largest possible radius that did not touch the eyelidsl for the maxillary regions we used two ellipses whose longer axis ran from nose to face boundaries, and shorter axis was half of the longer one. The ellipses were centered in the middle of the maxillary region. The average temperature for each subject per phase and per condition was normalized to the average of the correspondent fixation cross temperature (Phase 0, baseline) and used as a dependent measure (i.e. Participant 1 Phase 1 Temperature = [Participant 1 (Phase1 Temperature) – Participant 1 (Phase 0 temperature)]. All variables were normally distributed (Kolmogorov–Smirnov d < 0.37, p > 0.15), and therefore, comparisons were performed using parametric statistical test. However, being the sample a relatively small one, we also used a non-parametric bootstrap technique in order to have a more robust measure of our effects^63^. For 5000 times we randomly assigned each data to each condition, entered the data in the same 2 × 3 × 5 ANOVAs, computed the F for each main effect and for the interactions. Then, we compared our original F-values with the distribution under the null hypothesis of the bootstrap F-values. The bootstrap p-level was calculated as the proportion of bootstrapped F-values (included in the 95% confidence intervals) greater than the original F-value^64^.

## Additional Information

**How to cite this article**: Panasiti, M. S. *et al*. Thermal signatures of voluntary deception in ecological conditions. *Sci. Rep.* **6**, 35174; doi: 10.1038/srep35174 (2016).

## Figures and Tables

**Figure 1:**
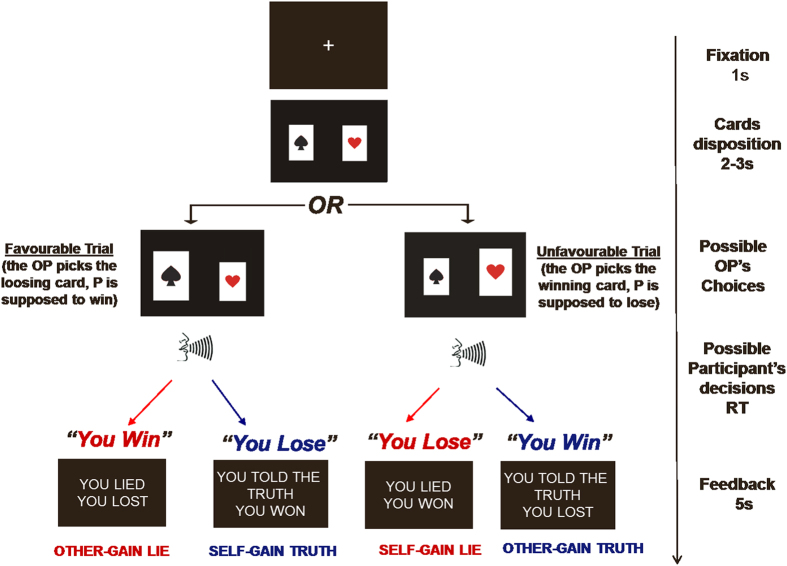
Schematic representation of the experimental procedure. Possible outcomes of the OP’s choice, possible responses of the P, feedback provided for each response, and possible categories of response. The time-line of the various phases is provided in the rightmost part of the figure.

**Table 1:** Mean (and standard errors) of the number of Self-gain and Other-gain lies produced in the two reputation conditions.

	Reputation Risk	No Reputation Risk
Self-gain lies	7.5 (1.01)	9.1 (1.04)
Other-gain lies	2.7 (0.99)	2.6 (0.81)
